# Tumorigenicity risk of iPSCs *in vivo*: nip it in the bud

**DOI:** 10.1093/pcmedi/pbac004

**Published:** 2022-02-03

**Authors:** Chaoliang Zhong, Miao Liu, Xinghua Pan, Haiying Zhu

**Affiliations:** Department of Cell Biology, Naval Medical University, Shanghai 200433, China; Department of Cell Biology, Naval Medical University, Shanghai 200433, China; Department of Biochemistry and Molecular Biology, School of Basic Medical Sciences, and Guangdong Provincial Key Laboratory of Single Cell Technology and Application, Southern Medical University, Guangzhou 510515, China; Shenzhen Bay Laboratory, Shenzhen 518032, China; Department of Cell Biology, Naval Medical University, Shanghai 200433, China

**Keywords:** induced pluripotent stem cells (iPSCs), tumorigenicity, regenerative medicine, reprogramming transcription factors, chemical-induced reprogramming, drug-inducible suicide system

## Abstract

In 2006, Takahashi and Yamanaka first created induced pluripotent stem cells from mouse fibroblasts via the retroviral introduction of genes encoding the transcription factors Oct3/4, Sox2, Klf44, and c-Myc. Since then, the future clinical application of somatic cell reprogramming technology has become an attractive research topic in the field of regenerative medicine. Of note, considerable interest has been placed in circumventing ethical issues linked to embryonic stem cell research. However, tumorigenicity, immunogenicity, and heterogeneity may hamper attempts to deploy this technology therapeutically. This review highlights the progress aimed at reducing induced pluripotent stem cells tumorigenicity risk and how to assess the safety of induced pluripotent stem cells cell therapy products.

## Introduction

Induced pluripotent stem cells (iPSCs), characterized by self-renewal and multiple differentiation potential, have been explored and applied in regenerative therapy, disease modeling, drug toxicity evaluation, and developmental biology. To date, transcription factor (TF)-mediated and chemical inductive reprogramming have been the strategies of choice to obtain iPSCs.^1–4^ Specifically, in TF-mediated reprogramming, the Yamanaka factors, Oct-4, Sox2, c-Myc, and Klf4 (OSMK), or alternative TFs (Table 1), are introduced into somatic cells with viral or non-viral vectors. In the chemical inductive reprogramming strategy, somatic cells are induced to become PSC through small molecules, cytokines, or growth factors.^4–6^ Romanazzo *et al*. summarized the merits and demerits of these reprogramming strategies and pointed out that the TFs used in the transgenic strategy lead to random epigenetic events, the activation of various pluripotent genes (i.e. Oct-4 and c-Myc), and genomic instability [e.g. chromosomal aberrations, copy number variations (CNVs), and single nucleotide variants (SNVs)].^7^ Such events were identified predominantly in iPSCs compared with embryonic stem cells (ESCs), suggesting a potential link between iPSCs and tumorigenicity. Compared with the transgenic strategy, chemical induction seems to decrease the possibility of tumorigenesis. However, the lower reprogramming efficiency and the induction system's complexity are still obstacles to the clinical application of chemical induction.

**Table 1.** Representative cocktail of TFs or TF-chemicals applied in the generation of iPSCs.

TFs	Chemicals	Efficiency	Original cell	Ref.
OSKM	/	0.02%	Human dermal fibroblasts	^8^
OSNL	/	0.022%	IMR90 fetal fibroblasts	^2^
OK & L-Myc	/	0.016%	MEF	^9^
OKM	/	0.001%–0.002%	Mouse neural progenitor cells	^10^
OSE	/	∼50% of OSKM	MEF	^11^
OSK	VPA	0.05%	MEF	^6^
OSK	/	<0.001%	Human dermal fibroblasts	^12^
OSNL	**/**	0.01%	Human newborn foreskin fibroblasts	^2^
OSK	VPA	1.1%	Human neonatal foreskin fibroblasts	^13^
OS	VPA	0.004%	Human neonatal dermal fibroblasts	^13^
OK	RepSox	0.05%	MEF	^14^
OSK	CHIR99021	0.2%–0.4%	MEF	^15^
O	VPA, HIR99021, RepSox	0.3%	MEF	^16^
SKM	VPA, SB431542	0.02%	MEF	^17^

OSNL: Oct4, Sox2, Nanog, Lin28; OSE: Oct4, Sox2, Esrrb; OSK: Oct4, Sox2, Klf4; OS: Oct4, Sox2; OK: Oct4, Klf4; O: Oct4; SKM: Sox2, Klf4, c-Myc; VPA: valproic acid; MEF: mouse embryonic fibroblasts.

Additionally, the heterogeneity of the cellular product derived from iPSCs for clinical therapy could lead to potential tumorigenicity risks *in vivo*. Theoretically, regardless of the strategy used to generate iPSCs, it may be impossible to avoid their heterogeneity due to the heterogeneity of the original somatic cells. Specifically, the established iPSC lines may still contain somatic cells and partially reprogrammed cells similar to iPSCs. Thus, the final cell preparation obtained may be a mixture of target cells for therapeutic use, the residual undifferentiated iPSCs, or partially differentiated iPSCs with teratoma-forming potential.^18^ The complicated *in vivo* environment makes cell integration and reproduction uncontrolled. Hence, decreasing the heterogeneity and increasing the controllability of iPSCs and cell preparation through isolation, purification, amplification, and modification of stem cells are necessary to decrease the tumorigenicity potential. Significant progress has been made over the last two decades in this research field, although many problems remain.

Finally, we focused on the current discussion of the safety assessment of cell therapy products (CTP) of iPSCs, including genome integrity, heterogeneity, and *in vivo* tumorigenicity. Although there are no mandatory provisions issued yet, the evaluation of iPSC genome integrity is recommended as one of the most important items because it presents a close association with the tumorigenicity of the iPSC products.^19^ To date, many alternative methods for checking genetic mutations have become available. However, the cost of the procedure, the complexity of results interpretation, and the workload of data analysis have to be considered when the practical methods are considered.^20^

## Optimizing the cocktail of reprogramming factors

As shown in Table 1, the past decade has seen the establishment of several combinations of TFs that can efficiently reprogram somatic cells based on the Yamanaka factors. Of these, c-Myc is the most controversial TF. It is well known that c-Myc is a proto-oncogene, encoding the family of beta helix–loop–helix/leucine zinc finger TFs,^21^ and its deregulated expression occurs in a wide range of human cancers, which leads to the discussion about the connection between c-Myc and iPSCs tumorigenicity. Hence, some researchers prepared iPSCs without c-Myc-based cell therapy to explore whether the absence of exogenous c-Myc can reduce iPSCs tumorigenic capacity without influencing the pluripotency.^9, 12, 22^ For example, Li *et al*. reported that 3-gene iPSCs (without c-Myc) differentiate into functional hepatocytes after receiving proper differentiation stimuli. iPSCs and iPSC-derived hepatocytes could decrease the thioacetamide-induced hepatic necrosis of mice and restore liver function in mice with lethal acute hepatic failure after undergoing intravenous or intrasplenic transplantation. This study highlights the potential of iPSCs, without c-Myc, in diminishing the incidence of tumorigenesis of cell transplantation.^23^

Alternatively, it is reported that c-Myc paralogs, such as L-Myc and N-Myc can be used to reduce teratoma in iPSCs.^21^ Nakagawa *et al*. chose L-Myc and c-Myc mutants (W136E c-Myc mutant and dN2 c-Myc mutant) to balance the efficiency and safety in reprogramming. Notably, compared to the mice derived from Myc-minus iPSCs, those from L-Myc iPSCs did not present higher tumorigenicity. Importantly, although the mice from L-Myc-iPSCs exhibited slightly higher mortality, these alternatives to the Myc family are worth considering when obtaining iPSCs with non/low teratogenicity from human somatic cells.^9^

In addition, some chemicals can replace specific TFs to help improve reprogramming efficiency,^14–17^ but the systematic studies on the tumorigenicity of iPSCs derived from different cocktails are not sufficient. Pushp *et al*. argued that cocktails containing TFs and small molecules are better in reprogramming efficiency than chemical cocktails alone, exhibiting less tumorigenicity than TFs only.^24^ The optimized formulas are listed in Table 1. For instance, Maherali *et al*. demonstrated that ALK4/5/7 inhibitor SB-431542 replacing exogenous c-Myc improved the reprogramming efficiency of mouse embryonic fibroblasts (MEF).^25^ Huangfu and colleagues discovered that valproic acid (VPA), a histone deacetylase inhibitor, and DNA methyltransferase inhibitors facilitate MEF and primary human fibroblast reprogramming processes.^26^, ^13^ Also, either RepSox or CHIR99021 could substitute for Sox2 and c-Myc,^14^, ^15^ and a combination of Oct4, VPA, CHIR99021, and RepSox could induce reprogramming of MEF to form alkaline phosphatase (AP)-positive clones.^16^ Unfortunately, there was no article showing results regarding the tumorigenicity of iPSCs derived from different cocktails.

## Strategy of chemical-induced reprogramming

Studies have shown that cell-fate reprogramming is comparably successful through the use of chemicals instead of conventional gene transformation.^4–6^, ^27^^–^^33^As listed in Table 2, Deng and his team reported a series of significant achievements.^4^, ^28^, ^34^, ^35^ Specifically, they obtained PSCs, named CiPSCs (chemically induced PSCs),^4^, ^28^ through chemical inductive reprogramming, but also obtained transdifferentiated cells directly from original somatic cells, known as direct reprogramming or lineage reprogramming.^28^, ^34^ In addition, recent reports demonstrated that biochemical signals (e.g. cytokines, growth factors, extracellular matrix (ECM) proteins, and small molecules) directly generated the target terminally differentiated cells from original somatic cells without making iPSCs in advance.^7^ Li *et al*. reported that the chemically induced extra-embryonic endoderm (XEN)-like cells obtained from MEF can be induced and reprogrammed directly to functional neurons and hepatocytes, bypassing the pluripotent state.^36^ Such findings might lead to a decreased tumorigenesis risk of iPSCs. However, a related systematic study on the underlying mechanism and tumorigenesis risk has not been reported to date. Moreover, unlike TFs, chemicals are mostly synthetic with clear targets for regulating biological activities, primarily through receptors and enzymes. More studies are needed to develop chemicals for reprogramming cell fate as reliably and rationally as TFs but without safety concerns. Chen *et al*. summarized the small molecule combinations that have been demonstrated with sufficient efficiency in somatic cell reprogramming and lineage reprogramming.^6^ It is worth noting that there are a few chemicals that promote reprogramming effectively through epigenetic modifications, such as histone deacetylase inhibitor TSA, SAHA and VPA, DNMT (DNA methyltransferase) inhibitor 5-AZA and RG108, histone methyltransferase G9a (Bix-01294) and H3K36 demethylase vitamin C.^6^ Fu *et al*. found that crotonic acid facilitated telomere rejuvenation through crotonylation and improved the generation of CiPSCs.^29^, ^37^ Considering the association between epigenetic alteration and tumorigenesis,^38^ it may be necessary to explore whether these types of compounds are safe or not in terms of oncogene activation or/and tumor suppression inactivation. Further, biophysical signals such as stiffness can also help achieve the direct reprogramming of somatic cells.^7^, ^39^

**Table 2.** Representative cocktails of chemicals applied in the generation of CiPSCs.

Source cell	Target cell	Small-molecule compound	Ref.
**Mouse fibroblast**	CiPSC	C6FZ (CHIR99021, 616452, FSK, DZNep) or VC6TFZ (VPA, CHIR99021, 616452, tranylcypromine, FSK, DZNep)	^4^
**Mouseintestinal epithelial cells(IECs)**	CiPSC	VC6TFZ (VPA, CHIR99021, 616452, tranylcypromine, FSK, DZNep) + AM580	^28^
**Mouseneural stemcells(NSCs)**	CiPSC	VC6TFE5Z (VPA, CHIR99021, 616452, tranylcypromine, FSK, EPZ, Ch55, DZNep)	^28^

Furthermore, although chemical cocktails work safely on mice, re-evaluation is needed in human cell reprogramming due to the differences between mice and humans in epigenetic memories and different pluripotent signal pathways.^30–32^ Up till the present moment, even though mouse CiPSCs have advanced in the last several years, generation of human CiPSCs has not been reported yet, which means a large-scale screening of small molecules may be necessary. That said, a few cases of successful lineage reprogramming with pure chemical compounds, of human somatic cells have been reported.^33^, ^35, 40–42^ However, the risk and efficiency for chemical induction of human cells remains to be explored case by case for each particular clinical protocol.^39^, ^43^

## Controlled mutagenesis of host gene caused by retroviral insertion and nanomaterial delivery system

Despite their robust efficiency, classical γ-retro- and lentiviral vector-based gene transductions have been linked with random insertions into the host genome, which may lead to unexpected genomic modifications. Therefore, several DNA-free strategies have been developed to circumvent the random integration of transgenes into target cell genomes.

Sendai-virus (SeV), a non-integrating adenoviral vector, has been demonstrated to reduce the possibility of genomic modification or gene silencing and derive integration-free iPSCs effectively because it has a complete cytoplasmic replication cycle.^44^, ^45^ Other non-viral methods, such as recombinant protein transduction,^46^, ^47^ repeated transfection with modified mRNA, and microRNA (miRNA), have also been tested. For example, the direct transfection of miRNA or the lentiviral expression of ESC-specific miRNA, such as *mir302* and *mir367*, can induce mouse and human somatic cells into iPSCs without introducing exogenous TFs.^48^ These DNA-free methods proved that transgene insertion is not essential for iPSC production and that an efficient decrease in the genomic modification risk can be obtained. Specifically, the footprint-free iPSCs generated by this method do not involve permanent genomic alterations and can also be differentiated into desired cells. However, these technically challenging methods are shown to be less efficient than retroviral transduction. Furthermore, they can only reprogram rare cell types such as fibroblasts; currently, this method may not be desirable when used to produce clinical-grade cells. However, most methods still represent options for *in vitro* biomedical applications, reducing the need for retroviral transduction.^49^

In addition, mRNA-based induction is a safe integration-free reprogramming method. However, due to the short half-life of mRNA and the obstruction of delivery, the efficiency of mRNA is lower than that of other methods.^50^ Recently, self-replicating RNA (srRNA), an improved synthetic modified mRNA-based method, was reported to be used in somatic reprogramming from human neonatal fibroblasts and was demonstrated to extend protein expression duration without risk of genomic integration. Steinle *et al*. believe that, due to its intergration-free properties, a single-shot of srRNA with higher reprogramming efficiency has good potential for application in the reprogramming research field.^51^

Moreover, delivery system is also one of the important factors influencing exogenous DNA integration. Although electroporation and chemicals are widely used, they may have the potential of reducing the cell activity or causing exogenous DNA integration. Nanomaterials, with the scale measuring ten to hundred nanometer, may offer alternatives to tranditional delivery methods because the higher interaction makes the stronger stimuli to cell feasible and the small nano-bio interface limits the extent of the perturbation and contributes to improve cell viability.^52^Wang *et al*. described a high-efficiency cellular reprogramming strategy achieved by puncturing cells with an array of diamond nanoneedles.^53^ This strategy achieves the delivery of mini-intronic plasmids (MIP) to generate iPSCs from human fibroblasts. The delivery process is finished within 5 min without cell lift-off. The efficiency is 1.17% $\pm $ 0.28% higher than traditional plasmid delivery methods. As an alternative method, the CRISPR/Cas9 system is usually delivered by plasmid, mRNA, or a ribonucleoprotein (RNP) complex, and has advantages of high efficiency and low off-target effects. However, RNP complexes do not performs well for cell reprogramming with multiple gene activation cell reprogramming. In contrast, magnetic molecularly imprinted polymers carry multiple RNPs and achieve high efficiency.^54^

## Eliminating teratoma-forming cells via the drug-inducible suicide system, small molecules, and immunodepletion

The heterogeneous somatic origin of iPSCs increases reprogramming uncertainty. Of note, when preparing cells for transplantation, the uncompleted reprogrammed cells, the undifferentiated iPSCs, and even the differentiated iPSCs may increase the oncogenic potential of therapeutic cells. This observation is a consequence of genetic or epigenetic aberrations from cellular reprogramming or prolonged cell culture.^26^, ^55^, ^56^ Therefore, for safe clinical application of iPSCs, it is essential to eliminate these cells during cell preparation. To this end, suicide gene technology is currently widely used to improve the safety of stem cell-based therapy. Specifically, the approach is to selectively eliminate aberrant therapeutic cells by activating a highly efficient safety switch. To date, three main suicide strategies have been developed.

One of these strategies is to use an anti-CD20 monoclonal antibody to induce antibody-dependent cytotoxicity, thus killing the cells expressing the B-specific human *CD20* gene exogenously. For example, Inrona *et al*. achieved the elimination of CD3 + CD20 + human T cells by adding monoclonal, chimeric anti-CD20 IgG1(kappa) Rituximab antibody (Roche) in the presence of complement. However, to date, no experiments have been performed applying CD20 to the iPSCs field.^57^

The second strategy is to use a metabolic suicide gene, herpes simplex virus thymidine kinase (HSV-TK) gene, and its prodrug, ganciclovir (GCV). The product of GCV yielded through phosphorylation by HSV-TK incorporates into replicating DNA, causing cell apoptosis. HSV-TK has been tested in human iPSCs as a suicide gene system.^58–60^ Studies have demonstrated that the HSV-TK-expressing cells can be eliminated both *in vitro* and *in vivo* with high specificity and efficiency.^59–62^ However, as Kimura *et al*. insisted, the HSV-TK system cannot sufficiently shrink the iPSC-derived teratomas *in vivo*.^58^ Furthermore, as a potent cytotoxic antiviral drug, GCV often unavoidably kills transplanted cells expressing the HSV-TK suicide gene system when used to treat herpes virus infections.^63, 64^

**Figure 1.**
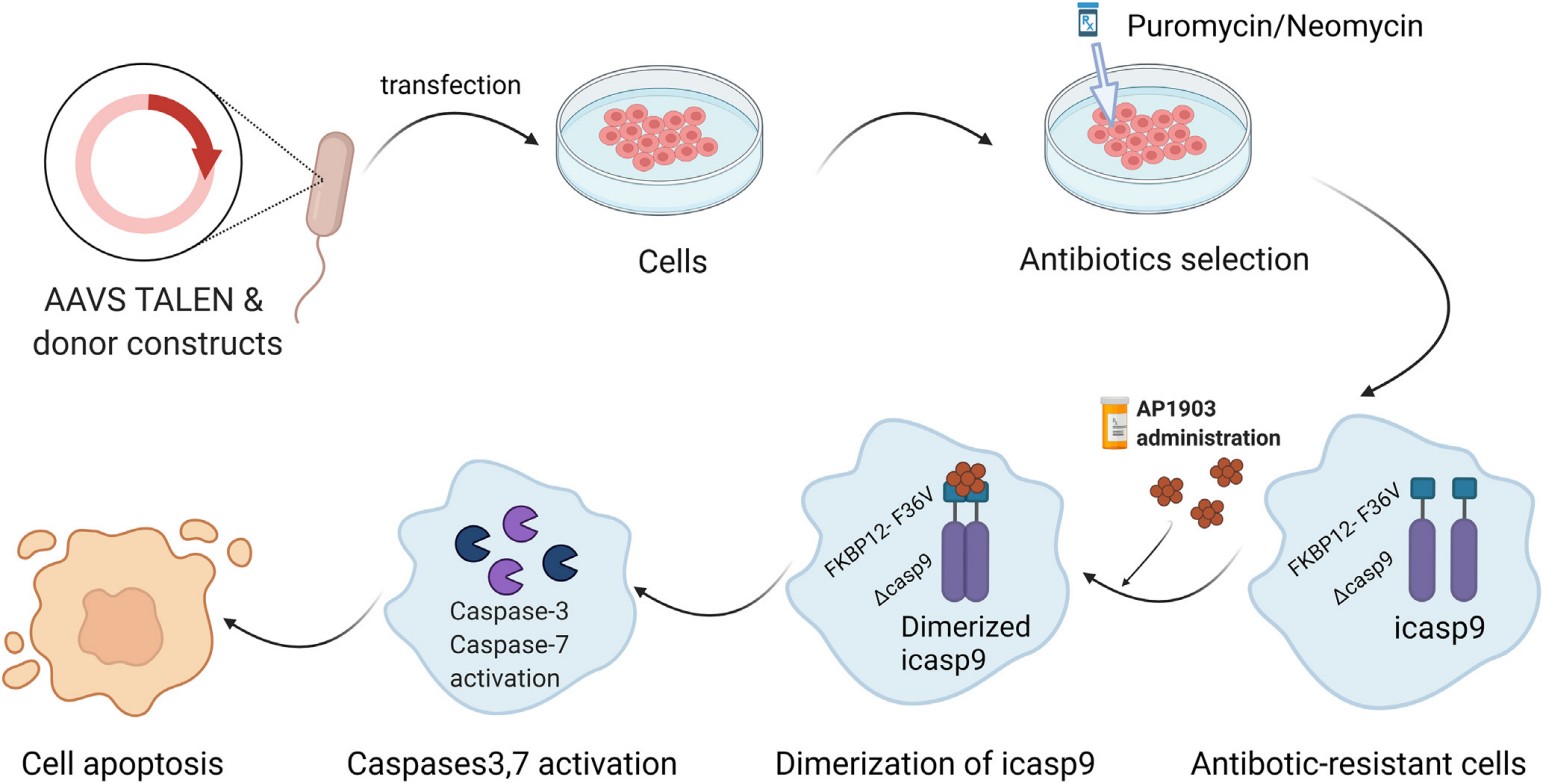
Mechanism of iCASP9’s safety switch. The AAVS1 TALEN and the donor constructs are delivered to cells via plasmid transfection. Then, the antibiotic-resistant cells are selected. Next, the administration of APs leads to the dimerization of FKBP12-F36V. As a result, caspase9 and the downstream effector caspases, such as caspase-3 and caspase-7, are activated, which leads to cell apoptosis.

The most recent suicide gene system, inducible caspase-9 (iCASP9), highlights the potential of iPSC-based regenerative therapy with improved safety.^65^ Its operating principle is to substitute the caspase recruitment domain of pro-apoptotic caspase-9 with a mutated dimerizer drug-binding domain from the human FK506-binding protein (FKBP12-F36V).^66^, ^67^AP1903 (aka rimiducid), a chemical inducer, binds to the F36V mutation with high affinity. Consequently, the dimerization of F36V, and the activation of caspase-9 and downstream effector caspases, such as caspase-3 and 7, occur (Fig. 1). Therefore, upon adding AP1903 in the medium, the iCASP9 system can induce apoptosis.^67^ This system is effective in lentivirus-infected T cells and human iPSCs.^68^, ^69^ However, the nonspecific lentivirus‐mediated genomic integration may lead to oncogenic, genetic changes, or unexpected silencing.^70^

To overcome the random genomic integration, the iCASP9-based lentiviral vector's genomic integration technique was improved to precisely introduce the gene into the genomic safe harbor AAVS1 locus. This resulted in the CAG promoter activating strong and stable expression of iCASP9. The AAVS1 locus resides in intron 1 of the PPP1R12C gene on human chromosome 19; this locus is widely used as an ideal and stable genome safe harbor site.^71^ The small molecule AP1903 could cause iCASP9 dimerization and cell apoptosis,^72,73^ eliminating iPSCs and iPSC-derived cells that integrated iCASP9. Of note, studies have shown that the iPSC-derived teratomas shrink dramatically upon applying AP1903.^63^

As an improved application in eliminating residual undifferentiated PCSs, Wu *et al*. selected the SOX2 locus as a safe harbor of the iCASP9 gene from three candidates, OCT4, Nanog, and SOX2.^74^ Specifically, the SOX2 gene had a lower risk of an off-target effect than the other two loci. Compared with the AAVS1 locus system, the SOX2i^CASP9^ system can precisely eliminate the iPSCs without affecting iPSC-derived cells without SOX2 expression. However, the SOX2^iCASP9^ system cannot be used to eliminate undifferentiated iPSCs mixed with cell types expressing high levels of SOX2, such as neural progenitor cells^75^ and liver progenitor cells.^76^ In contrast with that, NANOG expresses in rare differentiated lineages and has been applied as a safe harbor site by Martin *et al*. They engineered H9 hPSCs carrying three safeguard systems, NANOG^iCasp9^, ACTB^TK^, and ACTB^OiCasp9^ and demonstrated their efficiency in ablating undesirable cell populations upon small molecule (AP20187 and/or AP21967) administration both *in vitro* and *in vivo*.^18^

In addition, Lee *et al*. created another suicide system with a cytosine deaminase (CD) gene inserted within episomal vectors. CD converts non-toxic 5-fluorocytosine into 5-fluorouracil, which can kill cells expressing the CD gene. Furthermore, the transduced episomal vectors with *CD* genes in cells may be lost following extended cell passaging. This suicide system provided exogenous DNA-free iPSCs and exogenous DNA-free neural stem cells.^77^ This combination of exogenous DNA-free vectors and suicide genes may have broad application in the future.

To date, there are only a small number of alternative small-molecule-based suicide safety systems available for research and clinical cell-based therapies. Specifically, the iCASP9 suicide gene system has been demonstrated to be effective and safe in clinical trials.^66^ Table 3 summarizes the properties of suicide systems currently explored in the iPSCs field. Considering the required long-term safety of iPSC-based transplantation engrafted in the human body, it is necessary to develop new systems of “keys” (i.e. chemical inducers of dimerization) and “locks” (i.e. variations of the iCASP9-fusion protein) and evaluate the safety and efficacy of new combinations in the clinical application of iPSC-derived cell products in the future.

**Table 3.** Potential suicide systems applied in the iPSCs field.

Locks	Keys	Mechanism	Ref.
Herpes simplex virus thymidine kinase (HSV-TK)	Ganciclovir	Inhibition of DNA elongation	^58^
Inducible caspase-9 (iCASP9)	AP1903, AP21967, Ap20187	Apoptosis induction	^18^, ^63^
Cytosine deaminase (CD) gene	5-Flurocytosine	Inhibition of DNA elongation	^77^
CD-20	Rituximab antibody (Roche)	Ag–Ab binding reaction	^57^

In addition to the genetic methods, non-genetic means were applied to eliminating undifferentiated pluripotent cells. Chemical inhibitors of SURVIVIN, YM155,^78,79^ and cardiac glycosides, digoxin and lanatoside C^80^ were reported to selectively ablate undifferentiated pluripotent cells with no damage to the function and survival of differentiated cells. Immunodepletion is another effective strategy. Tang *et al*. used a cocktail of antibodies against anti-stage-specific embryonic antigen (SSEA)-5 and pluripotency surface markers to remove teratoma-formation potential and obtain purified differentiated cell cultures.^81^ Most recently, the function of monoclonal antibody K312^82^ and chimerised monoclonal antibody (mAb) ch2448, in depleting residual PSCs and preventing teratoma formation were reported.^83^ Of note, the premise of using this marker-based strategy is that the markers specifically expressed in PSCs so as to avoid killing differentiated cells while removing undifferentiated PSCs.^18^

## Maximizing the purity of iPSC samples

It is well known that fluorescence-activated cell sorting (FACS) and magnetically activated cell sorting (MACS) are used to efficiently isolate specific target cells from cell cultures.

**Figure 2.**
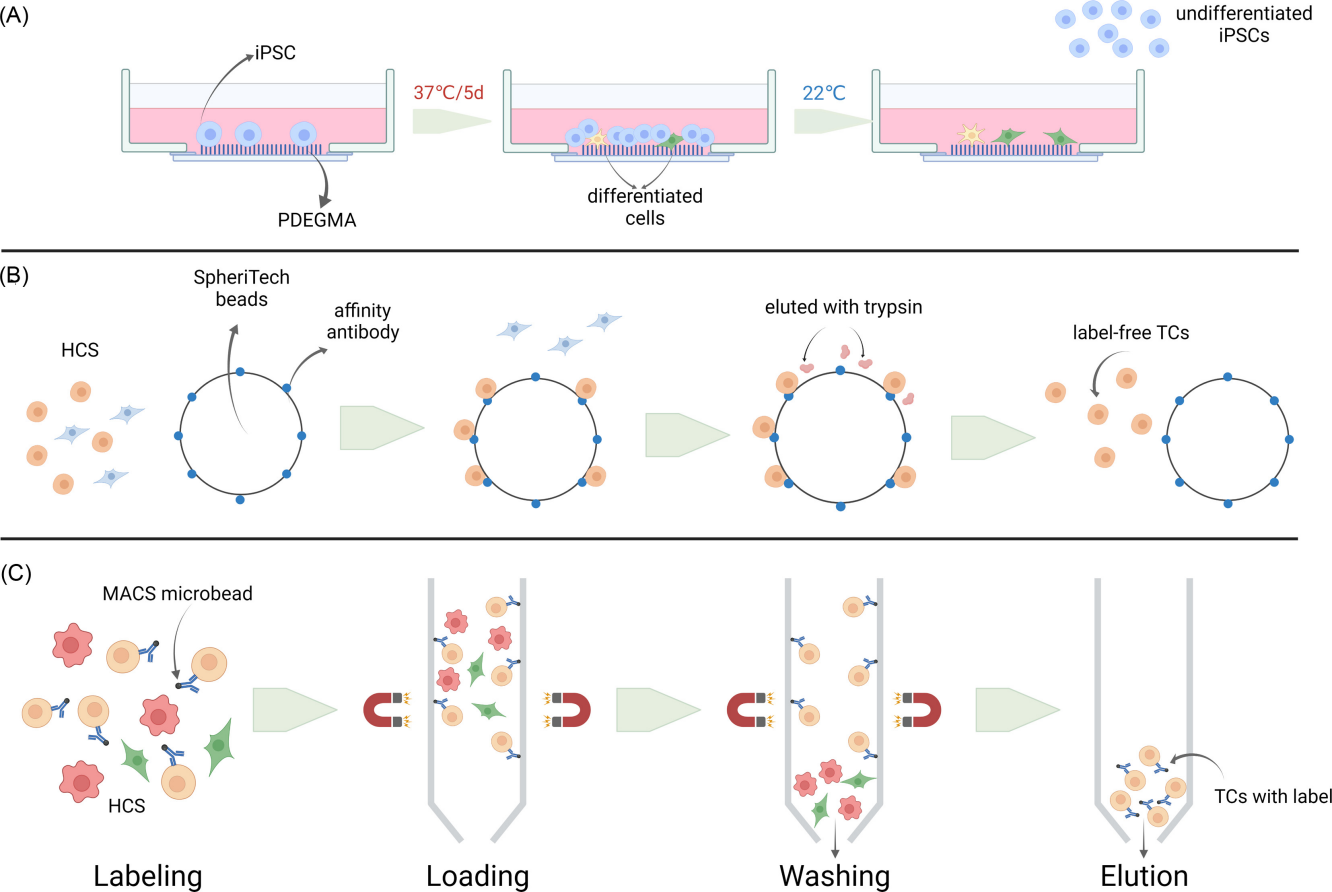
Schematic diagram of methods for purifying iPSC samples. (**A**) LCST behavior of PDEGMA. iPSCs are cultured on the polymer at 37°C for 5 days and grow into colonies on the layer. By cooling the temperature to 22°C, iPSC colonies detach from the layer while differentiated cells remain on the layer. (**B**) After incubation with SpheriTech beads coated with affinity antibody, the target cells in a hetergenous cell suspension bind to the beads. Label-free target cells are eluted from the beads by trypsin and affinity antibodies still stay attached to the beads. (**C**) Compared with SpheriTech beads, antibodies or magnetic particles attached to the target cells may influence the cells for positive selection by MACS. HCS: heterogeneous cell suspension; TCs: target cells.

Regularly, MASC is used to isolate iPSC-derived cells from derivation plates prior to transplantation. The main steps of MASC include labeling, loading, washing, and elution. Unlike somatic cells, iPSCs are usually passaged as clumps. Hence, it is necessary to obtain a single-cell suspension before the “labeling” step with specific antibodies. For example, Rho-associated protein kinase is used to dissociate iPSCs clumps (Fig. 2). However, this technique has been proven to reduce the viability of iPSCs. More recently, Gao *et al*. adapted the DEF-CS medium (iPSCs culture system from TaKaRa Bio USA) to obtain single iPSCs, achieving a >80% viability rate.^84^ Another problem requiring a solution is MACS’ limited efficiency in depleting undifferentiated iPSCs from a heterogeneous population of cells. The application of multiple magnetically labeled antibodies can improve the efficiency without affecting the viability. For example, TRA-1–60 or SSEA4 antibodies with MACS are useful for iPSC selection.^85–87^ As matter of fact, depending on the target cell type, specific surface markers have been successfully used for positive selection. For example, CD73+ photoreceptors have been isolated from iPSC-derived retinal organoids with high purity.^88^ In a separate study, neural progenitor cells were separated from neural crest cells by MACS with CD271 depletion, followed by CD133 selection.^89^ Highly efficient positive selection needs not only specific antigens but surface markers as well. It is therefore essential to select more specific surface markers on target cells to improve MACS efficiency.

Moreover, enrichment of label-free target cells from heterogenous cell cultures is required for clinical treatment. Recently, novel antibody-based beads, SpheriTech beads, have been applied to purify target cells without labels. The beads are paramagnetic and the affinity antibody is covalently immobilized onto their surface. Although the beads with cells need to be held and washed in magnetic field as MACS, it is the label-free target cells with high purification and activity that are finally eluted with trypsin and collected (Fig. 2B). Comparing with FACS and MACS, SpheriTech cell sorting exhibited lower expense and more simplified operation when it is used to sorting CD73-positive retinal photoreceptor progenitors from iPSCs induction.^90^, ^91^ Besides, a responsive polymer-modified system could be an option to achieve label-free cells. Jiang *et al*. have used this system to achieve label-free cell separation of iPSCs. Specifically, this system is based on the lower critical solution temperature (LCST) behavior of poly (di (ethylene glycol) methyl ether methacrylate) (PDEGMA), a thermo-responsive polymer used to make a homopolymer layer. For iPSC purification, the first step is incubating iPSCs at 37°C for 5 days to help them grow into cell colonies. Subsequently, the temperature is cooled to 22°C to promote the detachment of undifferentiated iPSCs from the layer, while differentiated cells remain attached (Fig. 2). The latter approach leads to the separation of iPSCs from differentiated iPSC-derived cells, keeping the viability of iPSC-derived cells and the pluripotency of iPSC at high levels.^92^

Except for antibody-based separation, specific chemical staining is available to sort iPSCs. AP with high expression in pluripotent stem cells, such as iPSCs, can hydrolyze phosphate in cells under alkaline conditions. Although AP staining is not a definitive standard for the established iPSC clones, the number of AP positive clones are applied to evaluate reprogramming efficiency.^93^ However, AP colonies stained with previous substrate cannot be propagated any further. Recently, an improved substrate, AP Live Stain, is being used to measure and visualize the kinetic process of somatic reprogramming. The stained iPSC colonies can be still further passaged and identified with additional specific markers because the characteristics and integrity of stained cells are not changed.^87, 93^

## Assessment of the quality of iPSCs before clinical use

It is realized that the safety and efficacy of iPSCs or iPSCs-derived cells must be evaluated before they can be used in clinical treatment. With an in-depth understanding of the proliferation and differentiation characteristics of iPSCs *in vitro*, researchers have put forward their own views regarding quality control and related methods.

Colter *et al*. noticed that the complexity of iPSC production and downstream inductive differentiation processes brought about uncertainty from processing to clinical effect, including low quality, heterogeneity and other problems. Therefore, the production and application of stem cells should rely on more rigorous and effective quality control of molecular and cell characteristics. It is necessary to establish a systematic evaluation of the variability between different batches of products and complete datasets combined with relevant computational methods. These efforts will help to develop a practical model and improve the robustness of the production process.^20^

Assou *et al*. believe that, compared with ESCs, iPSCs need additional mandatory quality control because of the possibly acquired genetic changes, such as aneuploidy and oncogene mutations (such as TP53), which directly affect the safety and efficacy of iPSCs and derivative products.^19^ Therefore, genome integrity testing should be used as a routine test item, and karyotype analysis should be a standard method of evaluation.^19^ Thus, it is logically reasonable that mutation screening should be applied systematically and corresponding judgment standards should be established. Concerning the methods, short tandem repeats (STR) analysis can be considered as an essential indicator of genomic integrity in addition to karyotype. Indeed, STR analysis is also recommended as a mandatory item to indicate the genomic integrity of iPSC establishment.^94^ It is emphasized that the STR profile of qualified iPSCs should be established in early passages and must match that of the cell donor.^94^ The ANSI/ATCC ASN-0002–2011 standard for the authentication of human cell lines requires at least eight core STR loci with an 80% threshold match. Kerrigan *et al*. increased the number of STR loci to 15 and Taylor believed 16 STR specific sites may be necessary for the plain identification even if iPSCs are generated from autologous somatic cells.^95^ Besides, fluorescence *in situ* hybridization (FISH), array comparative genetic hybridization (aCGH), and other microarray approaches, such as quantitative PCR (qPCR), SNP arrays, digital drop PCR (ddPCR), and next generation sequencing (NGS) were also used to assess insertion and deletion (indel), CNV, and SNV. Baker *et al*. evaluated a set of CNV and SNV determination methods for advantages and disadvantages.^96^ It is proposed that PCR technology and FISH technology are more suitable for detecting known frequent small fragment mutations, while aCGH and NGS fit more for large fragment analysis.^97^

However, when comprehensively considering cost, workload of data analysis, and complexity of result interpretation, ddPCR is commonly applied at present, and has high accuracy and relatively low cost compared with aCGH, NGS, and FISH technologies. Therefore, when evaluationg tumorigenicity of iPSCS and iPSCs-derived cells, the ddPCR of the known oncogene mutations and chosen targeted sequencing could be set as the top priority in analysis. Of course, because NGS is the whole genome at single-base resolution and can detect most genetic anomalies, depending on the sequencing depth, it may be considered when a high standard is preferred. However, it will be difficult for high-depth NGS to become a routine means of quality control before the sequencing cost is reduced to an affordable price.^19,98,99^

Rehakova*et al*. proposed a set of mandatory criteria and “for information only” tests, including differentiation, genetic stability, identity, vector clearance, morphology, pluripotency, robustness, viability, and histocompatibility, and proposed a set of testing methods corresponding to the current good manufacturing practices and regulations about production of clinical-grade iPSC and ESC lines.^94^ In addition, with regard to the detection of iPSC heterogeneity, flow cytometry analysis is a reliable method because it is timesaving and robust, and the results are quantitative and comparable among different laboratories. Baghbaderani *et al*. argued that for iPSCs, the release criteria for clinical use should contain >70% of cells positive for SSEA4, OCT3/4, TRA-1–60, and TRA-1–81 and < 5% of cells negative for CD34+.^100^ In addition, because single cell RNA-seq showed its extreme power in elucidating the heterogeneity of cellular population,^101^ exampled by hematopoitic stem cells and mesenchymal stem cells, it may be applied in evaluation of purifity of iPSCs and iPSCs derivatives when a protocol is set up for a clinical product.^102, 103^

Besides the *in vitro*assessment items mentioned above, it is suggested that *in vivo* tumorigenicity be included, because cellular behavior in the engrafted site may be one of the most direct pieces of evidence to confirm the clinical usability of an iPSCs cell therapy product (CTPs). However, it is difficult to standardize the experimental conditions, such as the selection of animal model, the number of inoculated cells, the study duration, and the site of transplantation. For instance, immunocompromised mice are selected to test tumorigenicity, but there is still no recognized standard on the number of animals and the controls that are required to demonstrate that the CTP is unlikely to form a tumor. With regard to the site of transplantation, CTPs are now inoculated into the clinical equivalent site intended for patients via the clinical route, though its theoretical foundation is still debated. Moreover, the reduced lifespan of the test animal compared to humans also constitutes a limitation for the long-term observation of tumor formation.^104^ Therefore, Sato *et al*. pointed out that although customized assays have been established to test different products on a case-by-case basis, it is essential to reach a global consensus on the standard of the test approach so that it can be applied to any relevant CTP.^105^

## Conclusions

**Figure 3.**
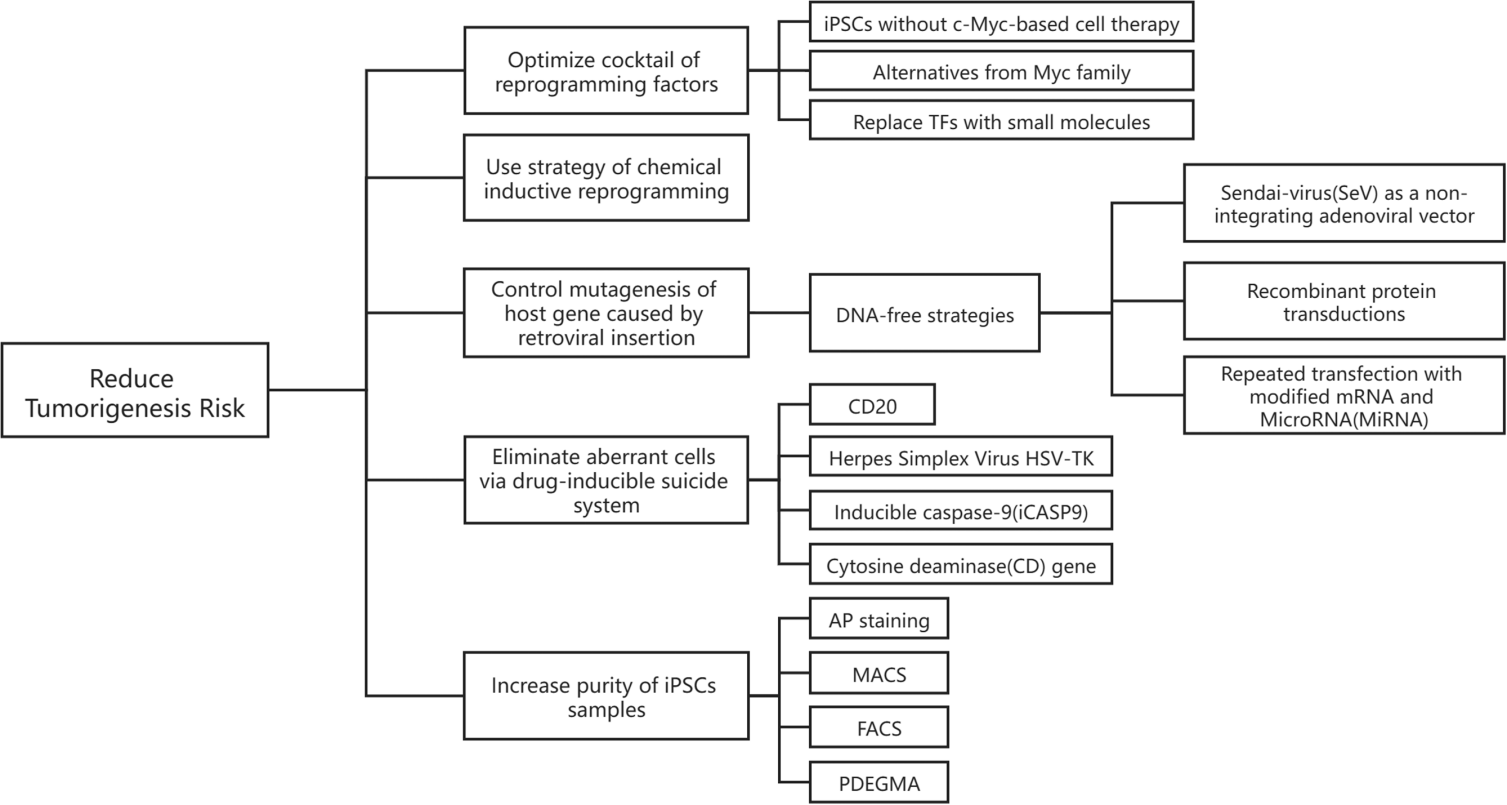
Strategies for reducing tumorigenesis risk.

This review focused on current progress to diminish the tumorigenesis risk of iPSC technology, including reducing the potential for tumorigenicity and promoting the killing of abnormal cells, as shown in Fig. 3. These efforts and further pursuits outlined here are critical for the clinic application of iPSCs technology.

A reduction in the tumorigenicity potential of the produced iPSCs can be obtained by optimizing the cocktail of reprogramming factors, using the strategy of chemical inductive reprogramming, purifying cells by FACS and MACS, and applying exogenous DNA-free vectors. Although the traditional MACS purification strategy is firmly established, a wider variety of iPSC surface markers, apparatuses, and protocols remain to be explored, such as SpheriTech beads, a novel label-free affinity purification method. In addition, interdisciplinary contributions like polymer materials and nanotechnology have shown potential to separate iPSCs and target cells, such as PDEGAM.

Currently, it is demonstrated that the suicide systems have very good application potential in effectively eliminating abnormal cells with teratogenicity in cell preparations of iPSCs. However, for improvement of specificity and efficiency, future studies are needed to design and develop new “keys” and “locks” and select specific safeguard sites for exogenous insertion.

Finally, systematic analysis of iPSC tumorigenicity, focusing on the mechanisms, is currently lacking. However, it represents an essential characteristic of iPSCs technology. Therefore, a standard of evaluation for cell preparation tumorigenicity in clinical applications should be established in the future, in which the genetic integrity validation of iPSCs and derivations is essential. In particular, assessment of safety seems to be more essential as more and more effective techniques, such as the CRISPR-dCas9 platform,^106,107^ are applied to fix the genetic defects of iPSCs derived from patients for subsequent therapy with the iPSCs CTPs in clinic.
